# Early outcomes of aortic valve neocuspidization (the Ozaki procedure): Initial experience of a single centre

**DOI:** 10.1016/j.ihj.2025.05.008

**Published:** 2025-05-24

**Authors:** Ranajit Naik, Abhishek Prabhu, Mohan Gan, Richard Saldanha

**Affiliations:** a Department of Cardiovascular & Thoracic Surgery, Jawaharlal Nehru Medical College, Belagavi, Karnataka, 590010, India; b Director, Adult Cardiothoracic and Vascular Surgery, MIOT International, Chennai, India

**Keywords:** Aortic valve neocuspidization, Ozaki procedure, Aortic valve replacement

## Abstract

**Introduction:**

Aortic valve neocuspidization (AVNeo) using fixed autologous pericardium, also known as the Ozaki technique, is an effective therapy for treating aortic valvulopathies. It serves as an alternative to complex aortic valve repair, offering better hemodynamics compared to biological or mechanical valve replacement, without the need for lifelong anticoagulation.

**Objectives:**

To evaluate the immediate and early outcomes of our initial experience with the AVNeo procedure in a spectrum of aortic valve diseases.

**Methods:**

In this retrospective cohort study, seventeen AVNeo procedures were performed between March 2023 and October 2023 at our center. All patients completed one year of follow-up. Outcomes were assessed by echocardiographic evaluation postoperatively and at one year, including complications and mortality.

**Results:**

Patient age ranged from 7 to 77 years (mean 35.2 ± 22.4 years), with 3 females among the 17 patients. Aortic valve morphology was bicuspid in 6 (35.3 %) and tricuspid in 11 (64.7 %) patients. Twelve patients had aortic stenosis as the primary pathology, and five had moderate to severe aortic regurgitation. In aortic stenosis patients, the preoperative mean peak gradient was 82.3 ± 20.3 mmHg, and mean gradient was 48.9 ± 18.5 mmHg. Postoperatively, mean peak gradient reduced to 9 ± 5.3 mmHg, and mean gradient to 16.7 ± 9.1 mmHg. Two patients required conversion to prosthetic valve replacement for progressive aortic regurgitation. There was no mortality at one year, though one patient developed severe aortic regurgitation.

**Conclusion:**

AVNeo is a feasible, reproducible procedure with favorable early outcomes, low pressure gradients, and mild regurgitation at one year. It is a cost-effective option, particularly in resource-limited settings like India.

## 1 Introduction

Aortic valve diseases are being increasingly diagnosed because of the increased use of echocardiography as a routine screening procedure for congenital as well as acquired heart diseases. Aortic valve diseases, both congenital as well as rheumatic, are being diagnosed early due to technological advancements. Valve replacement is the gold standard in the treatment of aortic valve pathologies. However, there is no ideal aortic valve substitute to date. An ideal valve prosthesis comprises optimized hemodynamics, low surgical mortality, long lasting durability, and long-term survival with good quality of life. Mechanical prosthesis implantation has limitations due to structural valve dysfunction and the need for lifetime anticoagulation. Bioprosthesis made of heterologous tissue undergo degeneration in 10–15 years among adults and more rapidly in younger patients. Thus the search for alternative options for aortic valve disease has increased in the last decade and includes repair, reconstructive and replacement choices.^1^, ^2^, ^3^

The innovative techniques being used are leaflet extensions, neo-leaflet creation, plication of prolapsing leaflets, commissuroplasty and transcatheter aortic valve implantation in high risk cases.

Despite the advances and innovations, treatment strategies available for children and young adults suffering from congenital aortic valve pathologies are limited and controversial. Though aortic valve repair is the most preferred and viable option in children and young adults, replacements may at times be required. In both adult and children, Ozaki or aortic valve neocuspidization procedure is emerging as a promising alternative.

Aortic valve neocuspidization was first described in 2011 by Professor Shigeyuki Ozaki in adult patients.^3^ It was approved by Food & Drug Administration, United States of America in 2014. The AV Neo system consists of single- or multi-use sizing templates, now marketed commercially by the Japanese organization of Medical Device Development company (JOMDD, Tokyo, Japan).

The original templates & sizers available were in sizes between 17 and 31 mm. Since 2015, pediatric sizers of 13 and 15 mm have also been available.

Despite advances in the design of valve prosthesis, hemodynamics performance and valve gradient values remain a concern in comparison to natural aortic valves. Ozaki technique offers hemodynamics comparable to the native aortic valve. Another advantage is the absence of lifetime anticoagulation because sutures are the only foreign materials used in this procedure. This new technique is applicable to both stenotic & regurgitant, aortic pathologies, including both tricuspid & bicuspid valves. It is also done in cases of infective endocarditis. There is no absolute contraindication for this procedure.^4^, ^5^, ^6^

It is used in both adult & pediatric population.^7^ In case where the autologous pericardium is thin or not durable due to pericarditis, the technique can be performed using heterologous pericardium.

The major concerns regarding this procedure include its long-term outcomes, training, and reproducibility. We have been performing the AV neo procedure in our patients since March 2023 following live case observation and dry lab training. In this study, we aim to report the early and immediate outcomes of our initial experience with the aortic valve neo procedure for a range of aortic valve diseases.

## 2 Methods

The study is an observational, single centre, retrospective study conducted at a tertiary care hospital in North Karnataka, India, between March-2023 and October- 2023.

All 17 patients who underwent aortic valve neocuspidization during the study period were included. Patients who underwent other types of aortic valve repair or single leaflet reconstruction were excluded. The study was approved by Institutional Ethical and Research Committee.

### 2.1 Data collection

Patient pre-operative records and postoperative follow-up data were recorded from the hospital medical records department and reviewed. The data relevant to our study were recorded in case report forms. The data collected included demographic characteristics, baseline pre-operative echocardiographic findings, intraoperative variables such as cardiopulmonary bypass time, cross clamp time, details of concomitant procedure details, operative time, postoperative echocardiographic findings, and complications including mortality.

### 2.2 Surgical technique

All operations were performed using autologous pericardium via median sternotomy with cardiopulmonary bypass. The neoaortic valve reconstruction with three leaflets was performed using glutaraldehyde-treated autologous pericardial cusps as defined by Ozaki et al.^3^ Autologous pericardium is soaked in glutaraldehyde solution for 10 min and rinsed three times in normal saline for 6 min each. The new cusps were created based on intercommissural measurements using the original device and templates marketed by the Japanese Organization for Medical Device Development (JOMDD, Tokyo, Japan). Del Nido cardioplegia solution was used for myocardial protection. The details of the concomitant procedures were also recorded.

In our experience, proper sizing with creation of appropriately sized cusps and commissural height play a crucial role in the success of the procedure. To achieve this, we recommend the using the meticulously designed templates designed by Ozaki et al.^3^^,^^4^ While some centres have attempted neocuspidization using alternatives, we believe Ozaki and his team have successfully standardized and made the procedure reproducible.

### 2.3 Postoperative anticoagulation

At our centre, all the patients undergoing Ozaki procedure receive initial anticoagulation with warfarin (target international normalized ratio: 1.5–2.5) and acetylsalicylic acid(75 mg) for 1 year, followed by long-term acetylsalicylic acid (75 mg).

### 2.4 Statistical analysis

The data collected were entered into a Microsoft excel spreadsheet. Statistical analysis was performed online using Social Science Statistics.

Continuous variables were expressed as means and standard deviations. The categorical variables were presented as numbers, frequencies and percentages.

## 3 Results

### 3.1 Demographics and baseline echocardiographic findings

The study included a total of 17 patients, of whom 14 (82.3 %) were male and 3 (17.7 %) were female. The mean age of the patients undergoing aortic valve neocuspidization was 35.2 ± 22.4 years as shown in Table 1.

**Table 1:** Demographics and baseline echocardiographic findings with diagnosis and valve morphology.

Characteristics	Total (*n* = 17)
Mean age	35.2 + 22.4 years
Gender	
Male	14 (82.3 %)
Female	3 (17.7 %)
Pathology	
Aortic stenosis	9 (52.9 %)
Aortic regurgitation	5 (29.4 %)
Mixed	3 (17.6 %)
Valve type	
Bicuspid	6 (35.2 %)
Tricuspid	11 (64.70 %)
Average pre-operative	
Mean gradient	48.9 + 18.5 mmHg
	82.3 + 20.3 mmHg

**Table 2:** Surgical and operative variables.

Mean Cardiopulmonary Bypass time	188.5 ± 23.7 min
Mean cross clamp time	154.35 + 22.6 min
Concomitant procedures	
Ventricular Septal Defect closure	01 (5.8 %)
Mitral Valve Repair	01 (5.8 %)
Decision for conversion to valve replacement	01
**Postoperative aortic regurgitation**	
No	03 (17.6 %)
Trivial	08 (47.05 %)
Mild	05 (29.4 %)
Moderate	00
Severe	01 (5.8 %) decided to replace
**Average gradient postoperatively**	
Mean	9 ± 5.3 mmHg
Peak	16.7 ± 9.1 mmHg

### 3.2 Diagnosis and morphology

The primary indication for aortic valve neocuspidization was aortic stenosis in 9 patients (52.9 %) and aortic regurgitation in 5 patients (29.4 %), while significant mixed lesion were seen in 3 patients (17.6 %) (see Table 2). The aortic valve morphology was bicuspid in 6 (35.3 %) patients and tricuspid in 11 (64.7 %). The average mean transaortic valve gradient was 48.9 ± 18.5 mmHg while the mean peak gradient was 82.3 ± 20.3 mmHg as shown in Table 1. None of our patients had a history of any prior interventional procedures.

### 3.3 Surgical and operative variables

The mean cardiopulmonary bypass time was 188.5 ± 23.7 min, while the aortic cross clamp time was 154.35 ± 22.6 min.

The concomitant procedures performed in the study group included closure of ventricular septal defect (*n* = 1), mitral valve repair (*n* = 1) and coronary artery bypass grafting (*n* = 1). The conversion to valve replacement was done in 1 case in view of residual moderate aortic regurgitation. The total of 7 paediatric patients were included in the study group. Among these two patients underwent aortic valve neocuspidization along with ventricular septal defect closure and mitral valve repair respectively.

### 3.4 Postoperative hospital course

In the immediate postoperative period, average mean transvalvular gradients was 9 ± 5.3 mmHg and mean peak gradient was 16.7 ± 9.1 mmHg. Trivial aortic regurgitation was observed in 10 (58.8 %), while 02 patients (11.76 %) had mild regurgitation, and 3 patients(17.6 %) had no regurgitation. No patients experienced severe aortic regurgitation in the immediate or early postoperative period.

However, 2 of our patients developed moderate and progressive aortic regurgitation during the immediate postoperative period. One underwent aortic valve replacement intraoperatively, and the other required valve replacement on postoperative day 1. Thus 2 patients (11.8 %) required conversion to aortic valve replacement.

The median intensive care unit stay was 4 days (range: 2–4 days) and the median postoperative hospital stay was 8 days (range: 7–10 days). One patient developed lower respiratory tract infection and another experienced mild wound soakage. No major complications such as bleeding, arrhythmia, or cerebrovascular events were noted during the immediate or early postoperative period (see Table 3).

### 3.5 One-year follow-up

All the patients completed one year of follow-up. At one year, the average mean aortic valve gradient was 9.1 ± 5.3 mmHg, and the average peak gradient at the end of one year was 15.3 ± 8.9 mmHg. Only one patient developed progression to severe aortic regurgitation during the follow up period and was advised further surgical management. All the other patients had trivial to mild aortic regurgitation at one-year follow up, as shown in Table 4. There was no incidence of AV block and any conduction abnormalities or infective endocarditis in our study during the follow up period.

**Table 3:** Post-operative complications.

No	14 (82.4 %)
Yes	3 (17.6 %) -Severe Aortic Regurgitation
Redo aortic valve replacement	01 (5.8 %) in view of severe Aortic Regurgitation
Respiratory tract infection	01(5.8 %)
Wound soakage	01(5.8 %)
Average postoperative hospital stay	8 days
Mortality	0 (0 %)

**Table 4:** Echocardiographic data at one year follow up.

Mean aortic valve gradient	9.1 + 5.3 mmHg
Peak aortic valve gradient	15.3 + 8.9 mmHg
Aortic regurgitation at one year	
No	06 (35.3 %)
Mild	07 (41.2 %)
Moderate	00
Severe	01 (5.8 %)

## 4 Discussion

The aim of our study was to analyze and report our centre's initial experience with Ozaki procedure or aortic valve neocuspidization, by assessing immediate and early outcomes of this procedure. Aortic valve neocuspidization has now been performed in both old and younger patients. Our study group included both young and elderly individuals, but primarily consisted of young adults. The early and immediate results of the study are favorable, with no mortality, acceptable low transaortic valve gradients, regurgitation, and minimal complications. The results align well with previous case series and larger studies reported globally. The 30 day mortality reported in other studies ranges from 0 % to 3.3 %.^8^, ^9^, ^10^ We observed no early or in-hospital mortality in our study group.

In this study, two patients required conversion to aortic valve replacement. One of these patients underwent reoperation on postoperative day one due to progressive moderate aortic regurgitation caused by commissural distortion. This may reflect the learning curve associated with procedure. Various studies have shown that aortic valve neocuspidization is a reproducible procedure but requires strict technical adherence.^11^^,^^12^, ^15^ These studies recommend that for surgeons who regularly perform aortic valve surgeries should complete at least 20 initial cases of neocuspidization to safely and effectively adopt the technique.^13^^,^^14^ With the exception of these two conversions, there were no major valve-related adverse events, such as bleeding or infective endocarditis, reported in our study. There was no requirement for pacemaker implantation, stroke intervention, or management of arrhythmia during the hospital stay. The incidence of pacemaker implantation following aortic valve neocuspidization has been reported, but remains lower than that associated with surgical or transcatheter aortic valve replacement.^16^, ^17^, ^18^

Aortic valve neocuspidization involves the implantation of newly constructed leaflets onto the annulus only without any upward force, making it suitable for patients with short annulus. Additionally, there is a lower risk of conduction block and thus a reduced need for permanent pacemaker implantation.

The mean and peak pressure gradients across the aortic valve reported in our study, 9 ± 5.3 mmHg and 16.7 ± 9.1 mmHg respectively are comparable to the results reported by Ozaki et al,^19^ Krane et al^13^ and the United Kingdom experience shared by Benedetto U. et al^11^

There was no significant difference in the transvalvular mean and peak gradients at one-year follow-up in our study. A total of 94.1 % of our patients did not require any intervention for aortic valve at one year. These findings are consistent with follow-up data reported by Benedetto U et al,^11^ Krane et al,^13^ Ozaki et al^19^ and Mylonas KS et al^28^

Aortic valve neocuspidization offers the advantages of very low postoperative gradients and a large effective orifice area. This benefit especially valuable in avoiding patient-prosthesis mismatch, particularly in individuals with a small aortic annulus. Some studies have observed that in such patients, the immediate post-procedure peak transvalvular gradients may be high, but hemodynamics tend to improve over time due to the remodeling of the pericardial cusps.^19^, ^20^, ^21^, ^22^

Although we did not encounter any cases of endocarditis in this series, it is hypothesized that the minimal use of foreign materials (limited to sutures) in Ozaki procedure may reduce the risk of infective endocarditis. For this reason, it may be preferable over mechanical or bioprosthetic valves in the setting of acute endocarditis. However, global experience in this context is still limited and requires further investigation.^23^^,^^24^

Aortic valve neocuspidization is both economically viable and technically reproducible. It is particularly attractive in resource –limited settings like ours, where financial constraints and poor anticoagulation compliance are common. In such contexts, Ozaki procedure offers a practical solution. It is generally recommended to use low-dose aspirin for six months after the procedure^4^. Some studies suggests lifelong aspirin use. Either approach is considered beneficial when compared to long-term use of vitamin K antagonists.

The initial experience at our centre reinforces the safety and the reproducibility of the procedure when performed by surgeon who routinely carry out valvular heart surgeries. From our perspective, aortic valve neocuspidization is significantly more complex than conventional aortic valve replacement and demands strict adherence to highly standardized operative protocol. As a result, it typically requires longer cardiopulmonary bypass and aortic cross clamp times. For isolated aortic valve neocuspization, cross-clamp times are reported to be approximately 100 min in multiple studies. In our study, the mean aortic cross clamp time was 154.35 ± 22.6 min. This may be attributed to the performance of concomitant procedures or the learning curve of the surgical team. Despite the longer cross-clamp and bypass times, the procedure did not lead to increased perioperative mortality or morbidity, a finding that is consistent with the results from other centres.

Aortic valve neocuspidization is increasingly seen as an effective alternative because it has very few absolute contraindications. The primary contraindications are related to damaged pericardial tissue, such as in patients with pericarditis, prior radiation therapy, or redo surgeries. In such cases, xenologous pericardium (bovine pericardium) or other commercially available material like PTFE may be used. However failure rates are higher when using these alternative materials, due to early calcification or tears at the suture lines.^24^, ^25^, ^26^

Infective endocarditis is one of the common indications for reoperation in long-term follow-up studies. In the original Ozaki series, the incidence of infective endocarditis was relatively high. This may have been due to extensive tissue handling during the early stages of the procedure's development. Autologous pericardium has shown greater resistance to bacterial infection compared to xenologous tissues. Some researcher hypothesize that using alternative materials may save surgical time, although this requires further evaluation.^5^^,^^24^

Aortic valve neocuspidization has emerged not only as a preferable superior alternative to bioprosthesis or mechanical valves in young adults but is also being considered as an alternative to the Ross procedure as well. Ross procedure carries significant risks, including mortality and later complications such as autograft dilatation and pulmonary dysfunction.^27^ Aortic valve neocuspidization can also be performed in patients with rheumatic heart disease both young and old, with favorable results. In cases where rheumatic heart disease affects more than one valve, the Ozaki procedure can be combined with mitral and tricuspid repair. This combined approach offers the benefit of avoiding lifelong anticoagulation, thus improving the quality of life. The procedure is an alternative and more affordable option compared to bioprosthesis valves, particularly in patients who are noncompliant or not willing to undergo lifelong anticoagulation. This makes it especially beneficial for pregnant women, young females who wish to complete their families, and patients with bleeding disorders.

Despite all the advantages, concerns about the long-term durability of aortic valve neocuspidization remain. There is a lack of extensive long-term follow-up data and randomized controlled trials assessing the longevity of this procedure. In their study with a mean follow-up period of 53.7 + 28.2 months, Ozaki et al^4^. reported freedom from death, reoperation and recurrent moderate regurgitation in 85.9 %, 4.2 % and 7.3 % respectively. A meta-analysis of reconstructed patient-level data by Mylonas KS et al reported one-year, three-year and five-year freedom from reoperation rates of 98 %, 97 % and 96.5 % respectively.^28^ As the midterm outcomes of Ozaki procedure are being established, an increasing number of centres are adopting it and reporting their experiences. We intend to follow up on our patients and report our outcomes in the future.

### 4.1 Study limitation

The present study is a small and retrospective, focusing on the initial experience at a single centre. Larger, multi-centre, prospective studies with longer follow-up periods are necessary to fully assess the long-term outcomes of aortic valve neocuspidization.

## 5 Conclusion

Aortic valve neocuspidization (AVNeo), utilizing autologous pericardium, is a promising and financially viable alternative for patients with a variety of aortic valve pathologies. Our initial experience demonstrates that the procedure is safe, reproducible, and yields favorable early and immediate outcomes, including low post-operative gradients and mild regurgitation. This procedure is an emerging alternative to aortic valve replacement with mechanical, bioprosthetic, or transcatheter valves—particularly in countries like India and other South Asian and African nations—due to its cost-effectiveness, lack of patient-prosthesis mismatch, and no need for anticoagulation, which benefits populations with limited resources and lower compliance.

## Ethical clearance

Obtained from Institutional ethical committee.

## Funding statement

No funding to declare.

## Declaration of competing interest

The authors declare that they have no known competing financial interests or personal relationships that could have appeared to influence the work reported in this paper.
